# Supplementary material for: Co-Promoted CoNi Bimetallic Nanocatalyst for the Highly Efficient Catalytic Hydrogenation of Olefins
Source: Nanomaterials (Basel). 2023 Jun 26;13(13):1939. doi: 10.3390/nano13131939 (PMC10343255; doi:10.3390/nano13131939)
Supplement: Supplementary file 1 [file nanomaterials-13-01939-s001.zip › nanomaterials-2300647-supplementary.pdf]

Supporting Imformation

Co promoted CoNi bimetallic nanocatalyst for the highly efficient catalytic hydrogenation of olefins

Fei Wu[1], Yueying Wang[2], Shunxin Fei[2*] and Gang Zhu[1*]

[1] Wuhan Institute of Marine Electric Propulsion, Wuhan 430064, China

[2] School of Materials Science & Engineering, Anhui University of Technology, Maanshan 243002, China;

* Correspondence:feishunxin@ahut.edu.cn; 180795242@139.com.

Computational Methods

In order to clarify valence electrons and core electrons, we chose the double-numeric polarization (DNP) basis set as well as the effective core potential [51,52]. All configurations were fully optimized, the charge transfer and distribution were performed by utilizing Mulliken population analysis [53]. Transition state (TS) search were carried out using LST/QST method [54].

The formation energy (ΔE_{FE}) was calculated by the following equation

$$\Delta E_{(FE)} = [xE_{Co} + yE_{Ni} - E_{CoxNiy}] / (x + y) \quad (1)$$

where m represents the maxmium H adsorption capacity at saturated state. Furthermore, ab initio molecular dynamics (AIMD) runs under 298.15K were performed using the Nose–Hoover thermostat for temperature regulate [55,56].

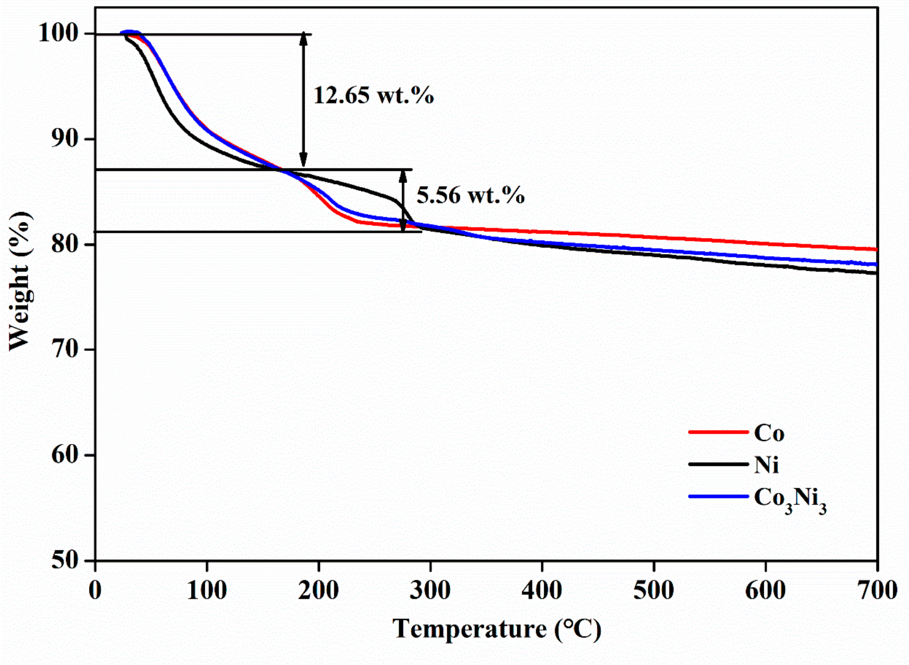

Figure S1. TG analysis of Co, Ni and Co_3Ni_3 precursors from 25 ℃ to 700 ℃.

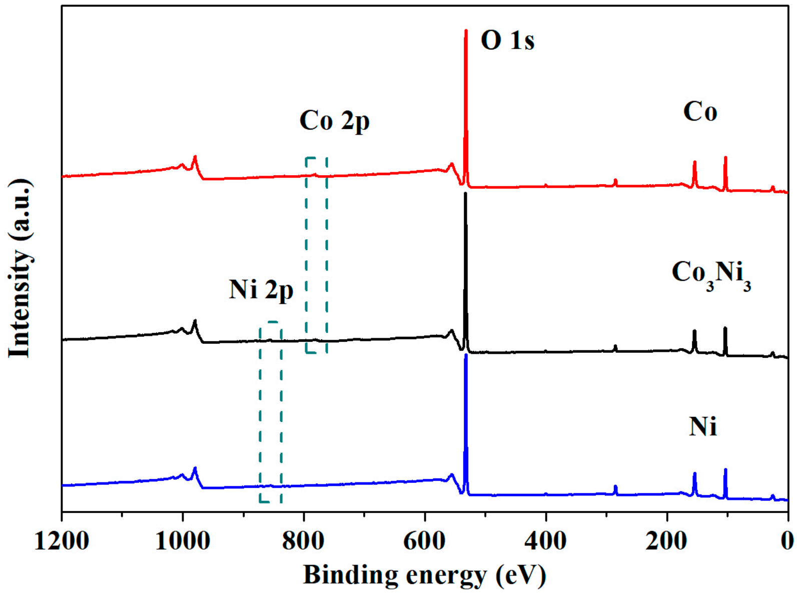

Figure S2. XPS full spectral analysis of pure Co/SiO_2, pure Ni/SiO_2 and Co_3Ni_3/SiO_2.

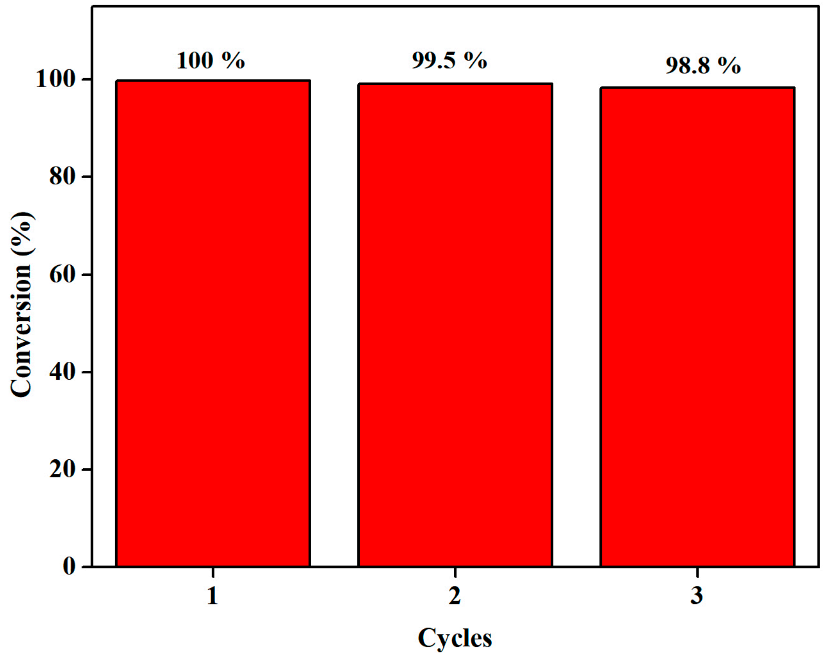

Figure S3. Catalytic cyclic hydrogenation of cyclohexene on Co_3Ni_3 at 100 °C, 2 MPa H_2 pressure in 90 min.

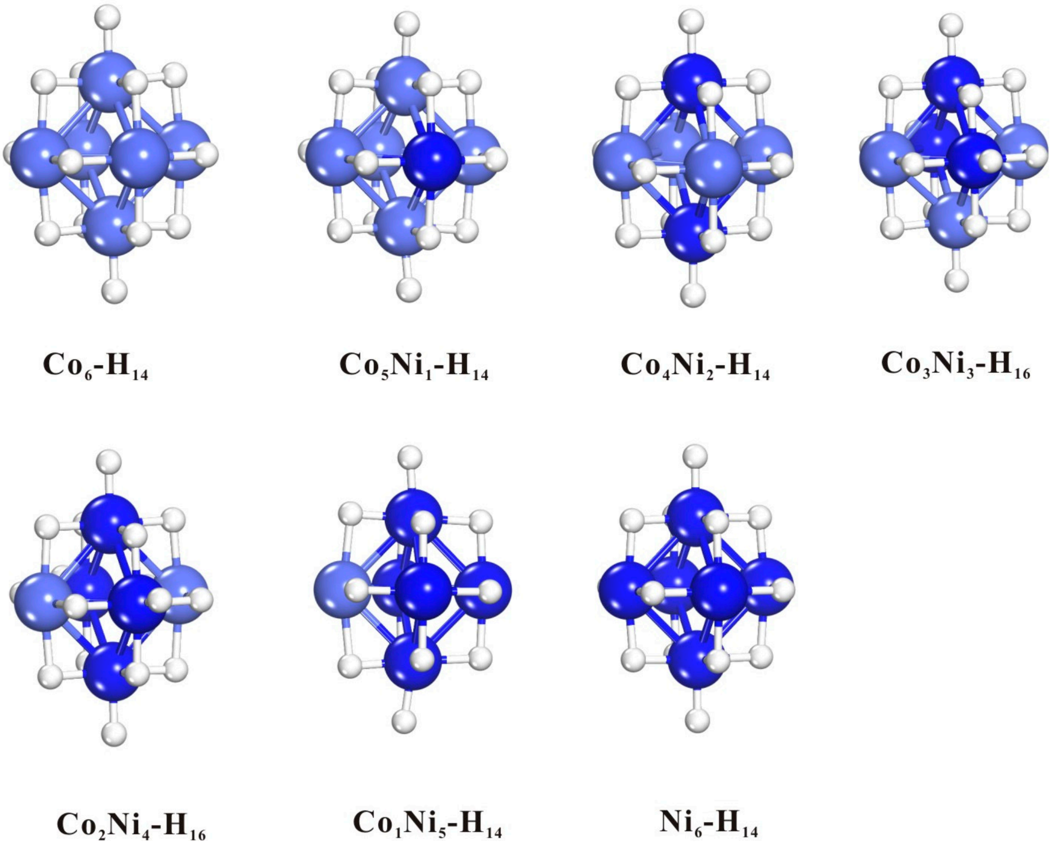

Figure S4. Optimized structures of Co_xNi_y clusters with maximum hydrogen capacity. Purple ball: Co atoms; blue ball: Ni atoms; white ball: H atoms.

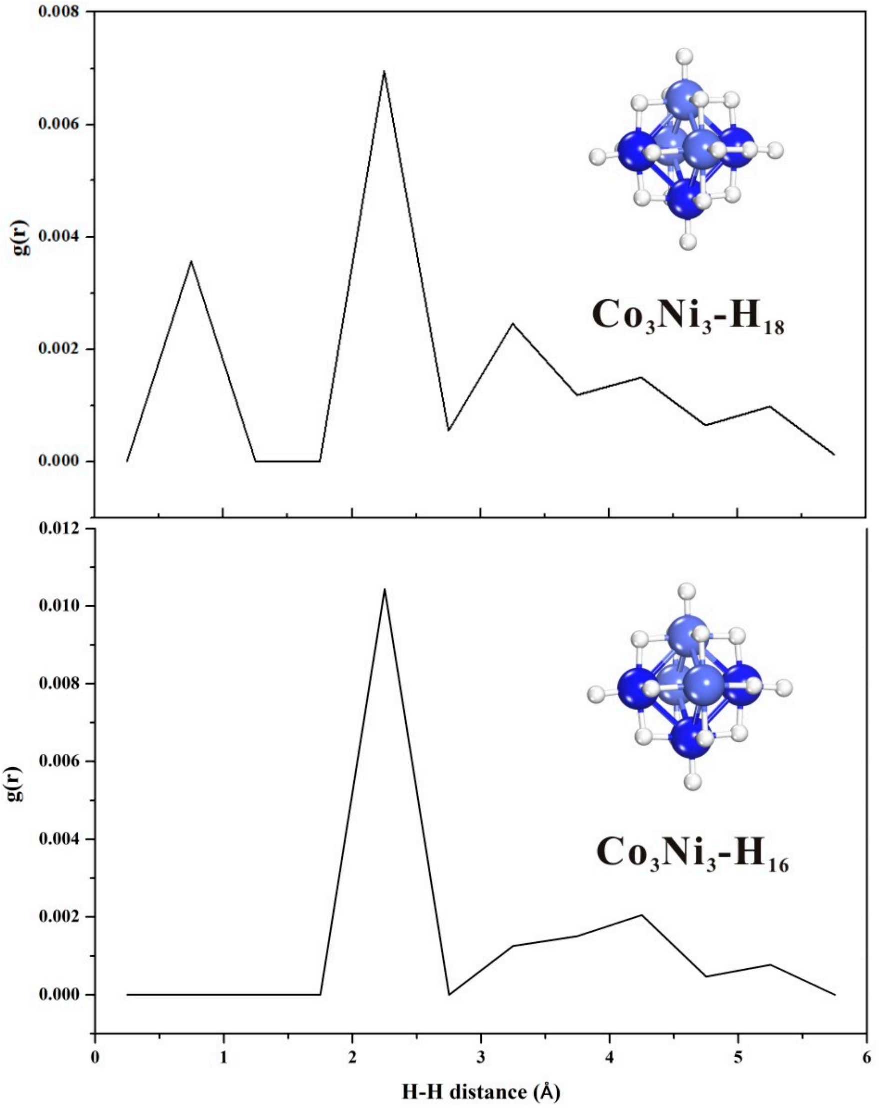

Figure S5. Simulated H–H distance distribution of Co_3Ni_3–H_{18} and Co_3Ni_3–H_{16} clusters. g(r) was made by tabulating all the data of H–H distances at every step of the AIMD trajectories fit with Gaussian functions.
